# LRPPRC-mediated folding of the mitochondrial transcriptome

**DOI:** 10.1038/s41467-017-01221-z

**Published:** 2017-11-16

**Authors:** Stefan J. Siira, Henrik Spåhr, Anne-Marie J. Shearwood, Benedetta Ruzzenente, Nils-Göran Larsson, Oliver Rackham, Aleksandra Filipovska

**Affiliations:** 1 Harry Perkins Institute of Medical Research and Centre for Medical Research, The University of Western Australia, Nedlands, WA 6009 Australia; 2 Department of Mitochondrial Biology, Max Planck Institute for Biology of Ageing, D-50931 Cologne, Germany; 3 Inserm U1163, Université Paris Descartes-Sorbonne Paris Cité, Institut Imagine, 75015 Paris, France; 4 Department of Medical Biochemistry and Biophysics, Karolinska Institutet, Stockholm, 17177 Sweden; 5 School of Molecular Sciences, The University of Western Australia, Nedlands, WA 6009 Australia

## Abstract

The expression of the compact mammalian mitochondrial genome requires transcription, RNA processing, translation and RNA decay, much like the more complex chromosomal systems, and here we use it as a model system to understand the fundamental aspects of gene expression. Here we combine RNase footprinting with PAR-CLIP at unprecedented depth to reveal the importance of RNA–protein interactions in dictating RNA folding within the mitochondrial transcriptome. We show that LRPPRC, in complex with its protein partner SLIRP, binds throughout the mitochondrial transcriptome, with a preference for mRNAs, and its loss affects the entire secondary structure and stability of the transcriptome. We demonstrate that the LRPPRC–SLIRP complex is a global RNA chaperone that stabilizes RNA structures to expose the required sites for translation, stabilization, and polyadenylation. Our findings reveal a general mechanism where extensive RNA–protein interactions ensure that RNA is accessible for its biological functions.

## Abstract (web summary)

The mitochondrial genome, being compressed to 16 kb, is an attractive model system to investigate how RNA-binding proteins chaperone mRNA lifecycles. Here the authors use RNase footprinting and PAR-CLIP to show that the LRPPRC–SLIRP complex stabilizes mRNA structures to expose sites required for translation and polyadenylation.

## Introduction

RNA-binding proteins (RBPs) regulate the lifecycles of RNAs from transcription to degradation and as such are important modulators of gene expression. This is particularly evident in mitochondria, where gene expression is predominantly regulated by nuclear-encoded mitochondria localized RBPs (mtRBPs)^1, 2^. The importance of nuclear and mtRBPs is exemplified by the number of different disorders caused by mutations in genes encoding these proteins, including neurological conditions, metabolic diseases, and cancer^3, 4^. Recent efforts have focused on transcriptome-wide identification of new RBPs in different cell types and organisms, as well as validation of already known RBPs or discovery of those with dual functions^5^. Consequently, an increasing number of these proteins have been assigned to different families/classes of RBPs, thought to play a role in RNA metabolism based on their sequence or fold homologies. Although we have insight into the RNA targets of a few well-characterized RBPs, the targets of most RBPs identified by high-throughput techniques are not known. Considering that RBPs coat RNAs extensively and thereby regulate their stability, translation and localization, it is essential to understand not only how they bind but more importantly the transcriptome-wide consequences of their loss in cells and organisms. This has been investigated at a genome level where loss of transcription factors, histones or histone modifying enzymes affect genome organization and transcription^6^, but this is lacking for post-transcriptional regulation of gene expression by RBPs both in the cytosol and mitochondria. Currently, the major goals in this field are the in-depth identification of RNA targets for RBPs and particularly characterizing their mechanism of action in vivo. Our study provides a model of transcriptome-wide consequences of mtRBP loss on RNA accessibility and secondary structure, revealing the critical role of a RBP as a RNA chaperone.

The sequence specific RNA-binding repeat proteins, including the Pumilio and FBF homology (PUF), and pentatricopeptide repeat (PPR) proteins, have been of particular interest because of their modular recognition of their RNA targets and their potential use as designer RBPs^7^. Although the RNA targets of PUF proteins have been identified and validated biochemically, structurally and in vivo, the physiological targets of PPR proteins have remained largely unknown, limiting our understanding of their mechanistic roles in organelle gene expression and energy metabolism^8, 9^. This is particularly true for the mammalian PPR proteins, where there are only seven PPR domain proteins that are all localized to mitochondria^10^. Cellular and in vivo studies have identified that these proteins regulate different aspects of mitochondrial RNA metabolism; from transcription via the mitochondrial RNA polymerase (POLRMT)^11, 12^, to RNA processing (PPR domain containing proteins 1 and 2, PTCD1 and PTCD2, mitochondrial RNase P protein 3, MRPP3)^13–19^, maturation and stability (the leucine-rich PPR cassette protein, LRPPRC)^20, 21^, and protein synthesis (the mitochondrial ribosomal protein of the small subunit 27, MRPS27, PTCD1 and PTCD3)^22, 23^. Although the RNA recognition code for plant PPR proteins has been identified computationally and using in vitro approaches^24^ and based on these findings artificial PPR proteins have been designed to modulate gene expression^25^, the RNA targets of mammalian PPR proteins remain elusive.

Here we use the mitochondrial transcriptome as a compact model to investigate and identify RNA–protein interactions at an unprecedented depth that has not been possible for the nuclear transcriptome. Mitochondrial genes coding for proteins and tRNAs are located on both the heavy and light strands of the mtDNA, and are transcribed as large polycistronic transcripts covering almost the entire length of each strand^26, 27^. This differs from nuclear gene transcription and requires several unique processing and maturation steps to form functional RNAs, all of which depend on mtRBPs. In nearly all cases, genes encoding protein or rRNA are interspersed by one or more tRNAs, which act as “punctuation” marks for processing^28^, recruiting the RNase P complex or RNase Z that carry out the cleavage at the 5′ end or 3′ end of tRNAs, respectively^15, 16, 19, 29^. Processing is followed by maturation of the RNAs by a range of mtRBP, including, the LRPPRC–SLIRP complex, poly(A) polymerase, methylases, tRNA modifying enzymes, and the CCA-addition enzyme, all necessary for the assembly of the rRNAs into mitochondrial ribosomes and translation of the mRNAs using tRNAs (reviewed in ref. ^1, 2^).

To understand the role of the LRPPRC–SLIRP RNA–protein complex in vivo, we sought to identify its RNA targets by photoactivatable ribonucleoside-enhanced cross-linking and immunoprecipitation (PAR-CLIP) and high-throughput RNase footprinting of mitochondrial transcriptomes from mice where each of the genes coding for these proteins are knocked out^20, 30^. LRPPRC was identified when a mutation in its gene was shown to cause a rare French-Canadian variant of Leigh syndrome with a cytochrome c oxidase deficiency^31^. LRPPRC is one of the longest PPR proteins known with 30–33 PPRs and does not contain any other functional domains^32^. LRPPRC forms a stable complex with SLIRP^20, 21^ via a PPR–RRM protein interface^32^, where SLIRP acts to protect LRPPRC from degradation in vivo^30^. Both proteins within the complex are required to regulate the rate of mitochondrial protein synthesis and the stability of the poly(A) tails of mitochondrial mRNAs^20^; however, the binding sites of the LRPPRC–SLIRP complex and its molecular role are not known.

Here we have investigated transcriptome-wide footprints of both LRPPRC and SLIRP, and the changes in molecular interactions and RNA secondary structure in their absence. We identify that single strand regions on mitochondrial mRNAs are the primary targets of the LRPPRC–SLIRP complex in vivo, indicating the role of this complex in RNA remodeling required to enable polyadenylation and translation. Furthermore we show transcriptome-wide changes in RNA interactions in the absence of these proteins, indicating that the LRPPRC–SLIRP complex acts as a global mt-RNA chaperone required to relax secondary structures. Our findings reveal the role of RBPs as chaperones for the lifecycles of mitochondrial mRNAs and provide insight into their recognition modes by investigating them at a much greater depth than previously possible.

## Results

### High-throughput identification of LRPPRC and SLIRP footprints

**Fig. 1:**
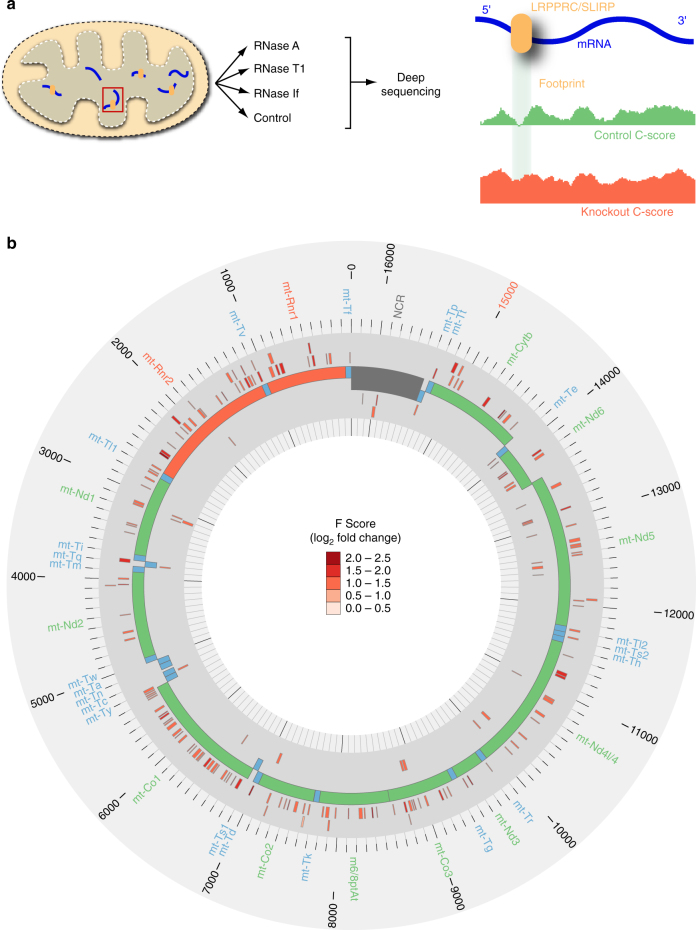
High-throughput RNase footprinting. **a** Schematic showing the principle of high-throughput RNA footprinting. **b** A circular representation of the mitochondrial genome (centre track) displaying LRPPRC footprints (exterior tracks) colored by the log_2_ fold change of F score (scale: 0.0–2.5). Control samples were scanned for footprints and compared to *Lrpprc* knockout samples to identify LRPPRC-binding sites. In the control samples we identify regions with F scores <2 that indicate lower RNase accessibility in the central footprint relative to the flanking regions. The control F scores are compared to the F scores identified for each footprint in the *Lrpprc* knockout samples and the significant log_2_ fold changes between the control and *Lrpprc* knockout mice are shown

To reveal the in vivo binding landscape of the LRPPRC–SLIRP complex we treated mitochondria isolated from hearts of conditional *Lrpprc* or whole body *Slirp* knockout mice, where these proteins are lost (Supplementary Fig. 1), with three endonucleases that have different cleavage specificities: (i) RNase A cleaves single- and double-stranded RNA after pyrimidine nucleotides, (ii) RNase T1 specifically cleaves single-stranded RNA after guanine residues, and (iii) RNase If, which is a sequence-independent endonuclease that preferentially cleaves single-stranded RNA over double-stranded RNA. The RNA isolated from these preparations was sequenced to enable transcriptome-wide in vivo mapping of footprints of each RBP. Mock-digested mitochondrial preparations were used as undigested controls. The data sets were aligned to the mitochondrial genome and normalized by sequencing depth; a cleavage score (C score) was determined for each nucleotide across the mitochondrial transcriptome for all data sets from coverage profiles of the 5′ nucleotide of each read, as previously described^33^. On the basis of these C scores, we identified regions within mitochondrial transcripts protected from endonuclease cleavage by LRPPRC or SLIRP in the control data sets. For each identified region, a footprint score (F score) was calculated based on the central RNase accessibility relative to the flanking regions in both controls (WT; *Lrpprc* ^*loxP/loxP*^ or *Slirp* ^*+/+*^) and knockout (KO; *Lrpprc* ^*loxP/loxP,Cre+*^ or *Slirp* ^*−/−)*^ data sets. A significant increase in the F score in the KO relative to the WT data sets enabled us to identify 178 different footprints for LRPPRC that were dispersed throughout mt-RNAs, the majority concentrated on mRNAs and to a lesser degree in rRNAs (Fig. 1, Supplementary Fig. 2 and Supplementary Data 1). There were fewer footprints in tRNAs and only two in the regulatory D-loop region of the mitochondrial genome (Supplementary Fig. 2), consistent with the reported roles of LRPPRC in mitochondrial mRNA polyadenylation and translation^20^. This is also in agreement with previous in vitro data where recombinant LRPPRC preferentially binds single-stranded mRNAs rather than tRNAs^32^. Most of the footprints were localized to mRNAs, and some LRPPRC footprints were particularly enriched across the entire length of transcripts such as the *Co1* mRNA, as well as *Atp8/6* and *Co3* mRNAs that often form a tricistronic transcript (Fig. 1). These findings suggest that LRPPRC binds regions of the mt-RNAs that are involved in processes such as RNA maturation, stability and translation. We observed extensive footprints along the two rRNAs, suggesting that LRPPRC may act as an RNA chaperone to facilitate rRNA processing and recognition by ribosomal proteins, since we now know that rRNA processing and ribosome assembly occur co-transcriptionally^19^. The identified footprints suggest that these sites are regulated by LRPPRC to enable their efficient maturation and recognition by the ribosome for translation. We analyzed footprints in the SLIRP knockout data sets to discover that there was no significant enrichment of footprints (Supplementary Fig. 3 and Supplementary Data 2), indicating that SLIRP does not bind RNA in vivo confirming previous in vitro biochemical findings^32^.

### An in vivo LRPPRC-binding signature identified by PAR-CLIP

**Fig. 2:**
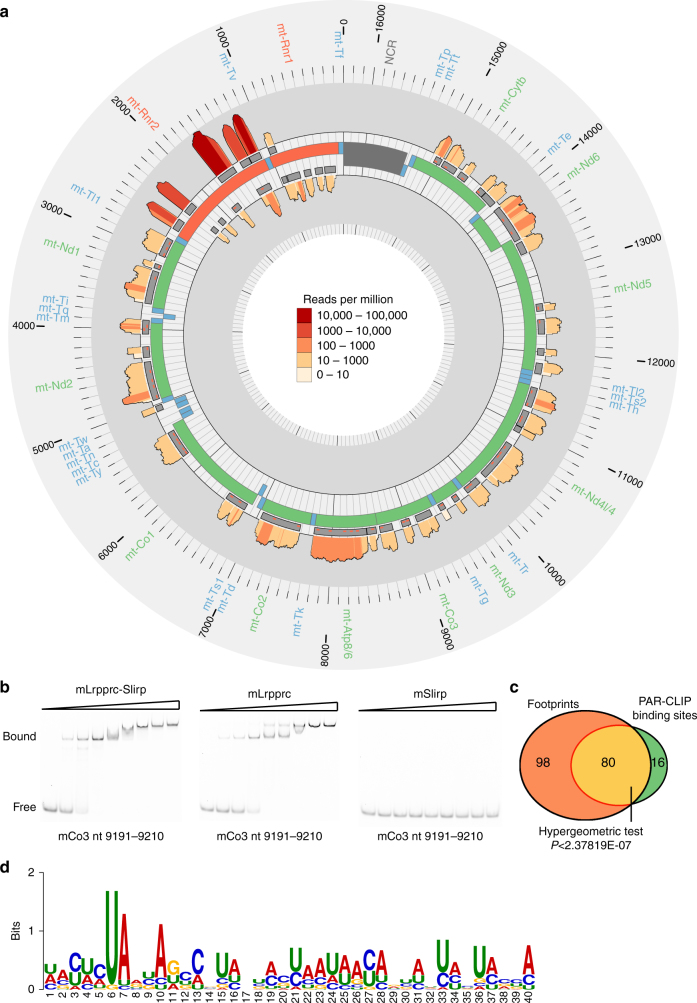
PAR-CLIP analysis of LRPPRC-binding sites. **a** Circular representation of the mitochondrial genome (centre track) displaying the PAR-CLIP-binding site regions (interior tracks) and normalized coverage (log_10_ (reads per million); scale: 1–100,000) across positions with at least a 95% posterior probability of being cross-linked (exterior tracks), as identified by BMix^50^. **b** RNA EMSA of the LRPPRC–SLIRP complex, LRPPRC only or SLIRP only incubated with a RNA probe identified as a strong LRPPRC–SLIRP-binding motif in vivo. **c** Schematic of shared binding sites among footprinting and PAR-CLIP data sets; the significance (*P*) of the overlap is indicated. **d** A sequence logo shows a predicted consensus binding motif for LRPPRC based on the binding sites that overlap between footprinting and PAR-CLIP data sets

We performed PAR-CLIP on mouse embryonic fibroblast (MEF) cells isolated from *Lrpprc-FLAG* transgenic mice to identify the direct binding sites of LRPPRC and to compare them with those identified by our footprinting. The incubation of MEF cells with 4-thiouridine (4TU) and its incorporation into de novo transcripts, followed by cross-linking and immunoprecipitation, enabled us to map the precise LRPPRC-binding sites by identifying sites with thymidine (T)-to-cytidine (C) transition rates indicative of cross-linking in the sequenced data set. The specificity of LRPPRC binding was established when compared to a control PAR-CLIP experiment that did not result in specific alignment to the mitochondrial transcriptome (Supplementary Table 1). We identified a number of direct and specific LRPPRC-binding sites, all located within mRNAs and rRNAs but not tRNAs, and the PAR-CLIP coverage across cross-linked sites is shown in Fig. 2a. Here again we find discrete binding sites of LRPPRC that are most often located throughout mRNAs, which require polyadenylation for stabilization and their coordinated translation, as shown before^20^. In the bicistronic mRNAs, *Atp8/6* and *Nd4l/4*, we found binding sites across the middle of the transcripts in the 3′ ends of the *Atp8* or *Nd4l* open-reading frames (ORFs) and the 5′ ends of the *Atp6* or *Nd4* ORFs, suggesting that LRPPRC may be required to expose the internal start and stop codons of these transcripts to the ribosome for translation initiation or termination. Interestingly, we found LRPPRC-binding sites within the 16S rRNA that were concentrated predominantly at either end of this transcript, suggesting that because of its affinity for transcript ends LRPPRC could facilitate rRNA processing, maturation and ribosome assembly. LRPPRC binding may facilitate the processing of the 16S rRNA from the RNA19 precursor transcript since loss of LRPPRC only decreases the levels of tRNA^Leu^ that is also contained within this precursor transcript^20^.

To validate the identified in vivo binding sites of this complex, we carried out RNA electrophoretic mobility shift assays (RNA EMSAs) using targets identified in both the footprinting and PAR-CLIP analyses. We show that the mouse LRPPRC–SLIRP protein complex has a high affinity for a region within the *mt-Co3* mRNA identified both in the footprinting and PAR-CLIP data sets (Fig. 2b) and that the recombinant mouse SLIRP alone does not bind RNA (Supplementary Fig. 4) as previously identified for human SLIRP^32^. Since the LRPPRC–SLIRP complex has high RNA-binding affinity with a large variety of RNA targets, we included a competitor RNA to mimic the physiological conditions in vivo. We show that the LRPPRC–SLIRP complex binds with higher affinity to specific regions within transcripts compared to other target regions of mitochondrial transcripts in vivo (Supplementary Fig. 5). The binding of these targets is higher compared to control or less enriched binding targets, indicating further that these are LRPPRC-binding sites. Nevertheless, we found that LRPPRC can bind to a wide variety of RNA targets in vitro and in vivo and might act as a general mRNA-binding factor in mitochondria, and that regions that are bound more frequently might be determined by a combination of binding affinity and accessibility.

Next we defined a set of high confidence direct RNA targets of LRPPRC by selecting a set of sites that overlap between our footprinting and PAR-CLIP data sets (Fig. 2c). The overlap between the two data sets was statistically significant and included the majority of PAR-CLIP sites (Supplementary Table 2). The direct binding sites were found predominantly in mRNAs and rRNAs, indicating that tRNAs are not direct targets of LRPPRC in vivo (Supplementary Fig. 6a). This was further confirmed by analyzing the footprints that did not overlap with the high confidence binding sites identified by PAR-CLIP, which were mainly distributed on tRNAs and rRNA (Supplementary Fig. 6b), indicating that these sites are protected from cleavage upon LRPPRC binding through the recruitment of other proteins or secondary structure changes. Motif searching using MEME identified a consensus binding sequence for LRPPRC (shown in red in Fig. 2a). These enriched sequences were somewhat degenerate (Fig. 2d), reflecting the preponderance of different sites recognized throughout the mitochondrial transcriptome.

### Transcriptome-wide consequences of LRPPRC loss

**Fig. 3:**
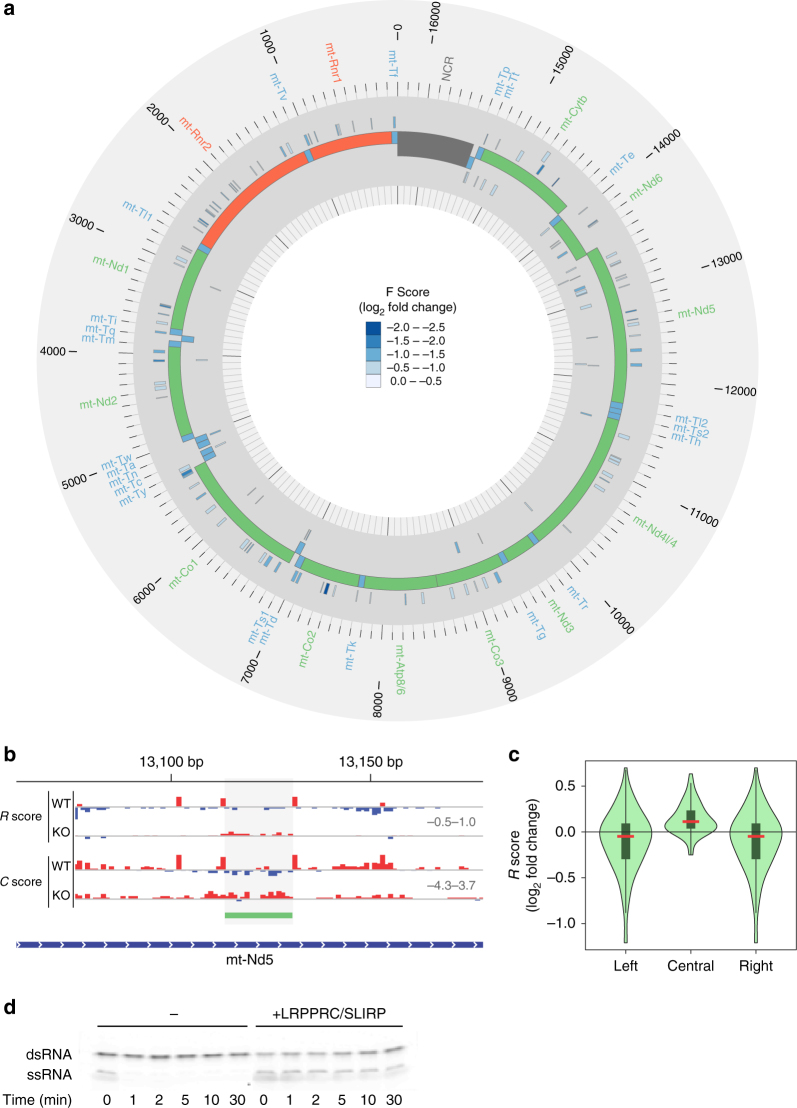
LRPPRC footprints show an increase in secondary structure propensity with its loss. **a** Circular representation of the mitochondrial genome (centre track) displaying footprints that are present in the *Lrpprc* knockout mice but not the controls (exterior tracks) colored by the log_2_ fold change of the F score (scale: 0.0–−2.5). The negative values indicate that we have compared the footprints present when LRPPRC is lost to control samples. **b** A footprint located in mt-Nd5, showing the increased C score (scale: −4.3–3.7) and R score (scale: −0.5–1.0) across the footprint region in the knockout. **c** Violin plot showing the distribution of the log_2_ fold change in average R score across all LRPPRC footprints that overlap PAR-CLIP-binding sites across the mitochondrial transcriptome, compared to the left and right 10 nt flanking regions. Loss of LRPPRC causes an increase in secondary structure propensity of mitochondrial RNAs. **d** LRPPRC demonstrates RNA chaperone activity in RNA annealing assays. Complementary oligoribonucleotides were hybridized in the presence or absence of the mouse LRPPRC–SLIRP complex

In contrast to the direct LRPPRC-binding sites, which are enriched in mRNAs and almost entirely absent from tRNAs, the footprints defined by comparing RNase accessibility in knockout and wild-type animals are found in all mt-RNAs, suggesting that the binding of LRPPRC affects the RNA structure and accessibility to other RBPs. Therefore to investigate transcriptome-wide consequences of LRPPRC loss we identified footprints in the LRPPRC knockout mice and examined their changes relative to control data sets (Fig. 3a). Here, we identified regions within mitochondrial transcripts protected from endonuclease cleavage by the absence of LRPPRC in the KO data sets. We identified 124 footprints with significant reduction of the F score in the knockout relative to the control samples, indicative of either RBP or secondary structure changes that render these specific sites resistant to RNase cleavage in the absence of LRPPRC (Fig. 3a and Supplementary Data 3). For example, we observe an appearance of a footprint in the *mt-Nd5* mRNA in the *Lrpprc* KO mice that may be a result of a change in secondary RNA structure or caused by another RBP that associates with this mRNA in the absence of LRPPRC (Fig. 3b). The number of footprints in the KO data set is less than the LRPPRC footprints identified in Fig. 1, suggesting that LRPPRC has a general RNA chaperone role in regulating RNA maturation, stability and translation. The same analyses in the SLIRP knockout data set failed to identify any significantly enriched footprints, further confirming that SLIRP does not exert any direct effect on RNAs.

Next, we investigated if RNA secondary structure can affect in vivo RBP interactions with the mitochondrial transcriptome in the presence and absence of the LRPPRC–SLIRP complex. We treated isolated mitochondria from control, *Lrpprc* and *Slirp* knockout mice with RNase If to specifically degrade single-stranded RNA and calculated an R score for each nucleotide across the transcriptome, where a higher R score is indicative of a greater propensity for strong secondary structure. We identified that loss of LRPPRC results in a net increase in the secondary structure of mitochondrial transcripts, whereas loss of SLIRP does not affect them (Supplementary Data 4). We examined the distribution of changes in average R score within footprints that overlapped PAR-CLIP-binding sites and identified that LRPPRC binding reduced the RNA secondary structure within its target sites (Fig. 3c). Furthermore, the average R score of regions flanking LRPPRC-binding sites was lower in the presence of LRPPRC (Supplementary Fig. 7a), therefore LRPPRC acts to prevent secondary structure formation where it binds and facilitates opening of flanking regions of RNAs, and preventing RNA within its binding site from interacting with complementary regions. We observed this effect of LRPPRC binding on mRNAs and rRNAs (Supplementary Fig. 7a–c).

To validate the in vivo finding that LRPPRC and the LRPPRC–SLIRP complex can act as RNA chaperones biochemically, we incubated purified proteins with complementary RNA oligonucleotides. Over time we observed that the presence of the LRPPRC–SLIRP complex blocked hybridization between the complementary RNA oligonucleotides (Fig. 3d). LRPPRC/SLIRP did not facilitate the annealing of complementary RNA oligonucleotides (Fig. 3d). This likely reflects that LRPPRC adheres to the canonical mode of RNA recognition of PPR proteins, where RNA adopts an extended conformation where the bases are recognized by direct hydrogen bonding interactions from consecutive repeats. To confirm this in vivo, we investigated the effects of LRPPRC loss on mitochondrial RNAs by RNA-Seq. We identify decreased polyadenylation of mitochondrial mRNAs (Supplementary Fig. 8a) and confirmed the reduction in poly(A) tail length in the absence of LRPPRC using modified 3′-RACE (Supplementary Fig. 8b). The loss of polyadenylation causes reduced stability of all mRNAs except *mt-Nd6* (Supplementary Fig. 9), confirming previous findings^20^, and corroborating that *Nd6* is the only mt-mRNA that is not polyadenylated (Supplementary Fig. 8a). Taken together, our results suggest that the LRPPRC–SLIRP complex acts to stabilize regions of single strandedness in mitochondrial RNAs and expose them for modification and maturation that enables coordinated translation.

### Conservation of LRPPRC–SLIRP binding in mouse and man

**Fig. 4:**
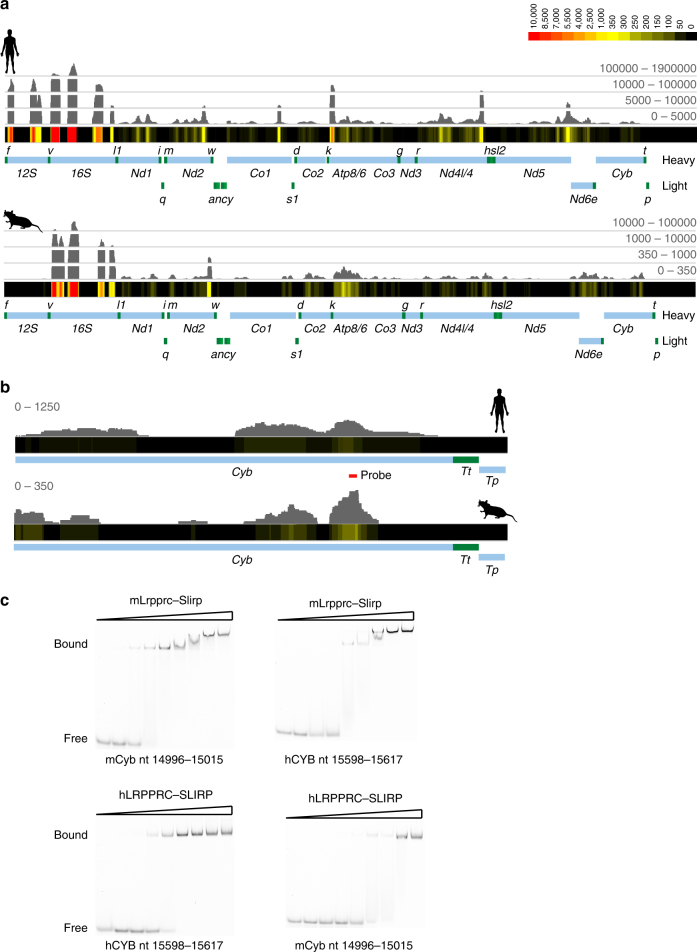
LRPPRC–SLIRP complex binding is conserved in humans and mice. **a** Transcriptome-wide distribution of binding sites in HeLa and MEFs expressing LRPPRC-FLAG determined by PAR-CLIP. **b** The locations of binding sites of the LRPPRC–SLIRP complex within *mt-Cyb* are well conserved in human and mouse. **c** RNA EMSA indicating the preference of the LRPPRC–SLIRP complex for its target within each species

Given LRPPRC’s broad substrate specificity, we wondered whether the locations of its binding sites were conserved through evolution. We performed PAR-CLIP in HeLa cells stably expressing LRPPRC that was FLAG tagged at the C-terminus and identified the binding sites of this protein in human mitochondria. The last common ancestor shared between human and mouse existed 96 million years ago^34^ and their mtDNAs are 70% similar at the nucleotide level such that most LRPPRC-binding sites vary in primary sequence between them. Nevertheless, we identified a very similar profile of binding sites when we compared the PAR-CLIP coverage profiles for LRPPRC in human cells to those identified in mouse cells (Fig. 4a). To determine if these similarities resulted from co-evolution of the LRPPRC proteins and their RNA targets or from restriction by other proteins or RNA secondary or tertiary structures, we assayed binding of RNA targets derived from a shared binding location in *mt-Cyb* that differed in sequence between human and mouse (Fig. 4b). We found that the mouse LRPPRC–SLIRP complex preferentially bound the mouse *mt-Cyb* RNA target, while the human LRPPRC–SLIRP complex preferred the human *MT-CYB* RNA target (Fig. 4c). These results show that the preferred locations of LRPPRC-binding sites within mitochondrial RNAs are constrained through evolution, likely illustrating their importance in dictating the local RNA structures that are critical in the lifecycles of mitochondrial RNAs.

## Discussion

The compact organization of the mitochondrial genome has resulted in a shift in the regulation of its transcriptome, from the transcriptional regulation of its proteobacterial ancestor to regulation predominantly carried out at the post-transcriptional level by RBPs, similar to gene regulation in the eukaryotic cytosol. Indeed, here we found that mt-RNAs were extensively bound by RBPs, as intimated by previous studies in human mitochondria, where the majority of the transcriptome was bound by mtRBPs^33^. As well as recruiting enzymatic activities and other protein complexes to RNAs, RBPs are often required to facilitate structural rearrangements of RNAs. The best characterized of these are the RNA helicases, that use ATP to unwind RNA helices with high processivity. The importance of RNA helicases is well established in processes such as RNA splicing, where a number of helicases are required to rearrange base paired structures between spliceosomal RNAs and their pre-mRNA targets. Less well studied, however, are ATP-independent RNA chaperones, whose activities do not require ATP, as they do not share a common evolutionary origin and rather are characterized by using strong direct interactions with RNAs to remodel their structures. Here we reveal that the LRPPRC complex fulfils this role in mammalian mitochondria.

LRPPRC-binding sites predominantly decorated the mRNAs and resulted in disruption of local mitochondrial RNA secondary structures. This suggests that relaxing the RNA structure by LRPPRC–SLIRP at these sites facilitates the coordinated translation and stability of mRNAs via polyadenylation^20, 35^. Also at the 3′ ends of mRNAs, LRPPRC–SLIRP could be required to present single-stranded RNA ends to the mitochondrial poly(A) polymerase (MTPAP) to facilitate polyadenylation. Evidence for this comes from in vitro experiments, where addition of LRPPRC increased the processivity of MTPAP^36^ and that loss of LRPPRC causes a polyadenylation defect^20^.

The mechanisms by which mitochondrial ribosomes recognize mRNAs and initiate translation are not currently understood. The start codons of mitochondrial mRNAs are typically found at the extreme 5′ ends with little or no 5′-UTR sequence. Our observation that the RNA chaperone, LRPPRC–SLIRP, relaxes RNA structures of mRNAs, may indicate that the mitochondrial ribosome requires the start codons to be exposed in order to initiate translation^20^. Furthermore, it is possible that LRPPRC’s chaperone activity might assist in the production of tRNAs. Since mRNAs are typically flanked by tRNAs, LRPPRC binding at the termini of mRNAs could prevent these flanking regions from interfering with tRNA folding, processing or modification. Indeed, previous analyses of the steady-state levels of tRNAs found that a proportion were altered in the absence of LRPPRC^20^.

LRPPRC has more PPRs than almost any other reported PPR protein, containing 33 predicted PPRs. Although structural information is only available for a few PPR proteins to date, it appears that they can often interact with their RNA targets in a binding mode where each PPR recognizes a single nucleotide base^37^. This might be important for LRPPRC’s ability to bind many diverse RNAs, since only a subset of repeats would need to recognize individual RNA bases in order to achieve sufficient binding affinity for each individual RNA target. Nevertheless, the specificity of LRPPRC’s PPRs is still important to target critical regions of the transcriptome, as exemplified by the co-variation we observed between LRPPRC and its RNA targets through the evolution of human and mouse.

Here we have shown that loss of a global RNA chaperone can lead to remodeling of secondary RNA structures as well as other RNA–protein interactions. Studies of the RNA-binding sites of eukaryotic nuclear and cytosolic RBPs have revealed sparse distributions of binding sites across individual mRNAs. However, given that there are an estimated 1542 human RBPs^38^, as an ensemble they likely act as LRPPRC does in the mitochondrial transcriptome to coat RNAs and assist in their folding. Therefore, the binding of LRPPRC across the mitochondrial transcriptome represents a minimal system to study the importance of ATP-independent protein-mediated RNA folding. The massively deep coverage of the mitochondrial transcriptome in our sequencing experiments has enabled us to elucidate the in vivo binding modes of the LRPPRC–SLIRP complex and establish a model approach of how to investigate the effects of RBP loss on a global transcriptome-wide level.

## Methods

### Animals and housing

*Lrpprc* and *Slirp* knockout transgenic mice on a C57BL/6 N background were housed in standard cages (45 × 29 × 12 cm) under a 12-h light/dark schedule (lights on 7 am to 7 pm) in controlled environmental conditions of 22 ± 2 °C and 50 + 10% relative humidity and fed a normal chow diet and water were provided ad libitum. LRPPRC loss is embryonic lethal therefore there are no full-body *Lrpprc* knockout mice and we used heart and skeletal muscle-specific knockout mice^20^. Also, SLIRP loss is not embryonic lethal and consequently we used hearts from the full-body *Slirp* knockout mice^30^. All mice used in this study were 12-week-old males. The study was approved by the Landesamt für Natur, Umwelt und Verbraucherschutz Nordrhein–Westfalen and performed in accordance with the recommendations and guidelines of the Federation of European Laboratory Animal Science Associations (FELASA).

### Mitochondrial isolation and RNase treatment

Mitochondria were isolated from homogenized hearts and isolated by differential centrifugation^30, 39^. Hearts or livers were cut and washed three times with ice cold PBS, and once with mitochondrial isolation buffer (MIB) containing 310 mM sucrose, 10 mM Tris-HCl and 0.05% BSA (w/v) by centrifugation at 4500 *g* for 1 min at 4 °C. Heart pieces were homogenized in 5 ml of fresh MIB using a Potter S pestle (Sartorius). The homogenate was centrifuged at 1000×*g* for 10 min at 4 °C and the supernatant was centrifuged at 4500×*g* for 15 min at 4 °C to isolate mitochondria. Crude mitochondrial pellets were suspended in MIB supplemented with 1x Complete EDTA-free protease inhibitor cocktail (Roche). Protein concentration was determined by the Bradford or BCA method using BSA as a standard. Mitochondria (2 mg ml^−1^) were lysed by addition of 200 µl of lysis buffer (100 mM Tris-HCl, 100 mM NaCl, 40 mM MnCl_2_, 2 mM dithiothreitol pH 7.5, 0.1% TritonX-100). The concentration of the RNase A (10 U µl^−1^), RNase T1 (0.1 U µl^−1^) or RNase If (0.01 U µl^−1^) were optimized and added to each mitochondrial lysate or purified RNA to generate 15–55 nt size fragments. All incubations were carried out at 37 °C for up to 30 min and reactions were ended by addition of 700 µl Qiazol, followed by RNA isolation using the miRNeasy Mini Kit (Qiagen). We used mitochondria lysed and mock treated as controls for the footprinting assay.

### Immunoblotting

Immunoblotting was carried out on mitochondrial proteins isolated from mouse hearts using a rabbit polyclonal antibody against LRPPRC (sc-66844, Santa Cruz Biotechnology, diluted 1:1000) and in-house monoclonal antibody directed against mouse SLIRP (diluted 1:200)^30^, in Odyssey Blocking Buffer (Li-Cor). IR Dye 800CW Goat Anti-Rabbit IgG or IRDye 680LT Goat Anti-Mouse IgG (Li-Cor) secondary antibodies were used and the immunoblots were visualized using an Odyssey Infrared Imaging System (Li-Cor).

### Library construction

RNA concentration, purity and integrity were confirmed by BioAnalyser (Agilent). The libraries were constructed using the Illumina TruSeq Small RNA Sample Prep Kit and deep sequencing of the mitochondrial small RNAs was performed by Australian Genomic Research Facility (Melbourne, Australia) on an Illumina GAII (Illumina) according to the manufacturer’s instructions with one modification, sample isolation from the PAGE gel after adaptor ligation was performed with a modified set of size markers to facilitate capture of small RNAs between 15–55 nt.

### Mapping and identification of footprints

Technical replicates were pooled and sequenced reads trimmed of adapter sequences with cutadapt v1.10, using default parameters, and aligned to the mouse genome (mm10) with Bowtie2^40^ v2.2.9 with a seed length of 10 and reporting up to 20 alignments per read (–L10 –k 20). Paired alignments with multiply mapping reads that aligned once to the mitochondrial genome and at least once to a NUMT region were rescued. All properly paired alignments to the mitochondrial genome with an observed template length of 15–35 nt were retained and a subtractive alignment against nuclear tRNA and Illumina contaminant sequences was performed. Strand-specific fragment BED files were created and 5′ coverage profiles normalized to sequenced library size were produced with BEDtools^41^ v2.26.0. Footprints were identified as previously described^33^ with some modifications reported here. The RNase accessibility of each base, i, in the mitochondrial genome was quantified according to its C score, defined as:

$${\it{C}}{\rm{scor}}{{\rm{e}}_{\it{i}}} = {{\log}_{10}}\left( {\frac{{{\rm{max}}\left( {{{\rm{A}}_{i + 1}},\,{\rm{T}}{1_{i + 1}},\,{\rm{I}}{{\rm{f}}_{i + 1}}} \right) + 1}}{{{\rm{Untreate}}{{\rm{d}}_{i + 1}} + 1}}} \right),$$

where A_*i*+1_, T1_*i*+1_, If_*i*+1_ and untreated_*i*+1_ represent the normalized 5′ coverage of the nucleotide immediately downstream of the inspected position in RNase A-, T1-, and If-treated and untreated samples, respectively. The footprint detection algorithm searches for a span of between 8 and 40 nucleotides with an average C score lower than the flanking left and right three nucleotides, the central footprinting region, and calculates its F score:

$$F{\rm{score}} = \frac{{{{10}^C}}}{{{{10}^L}}} + \frac{{{{10}^C}}}{{{{10}^R}}},$$

where *C*, *L* and *R* are the average *C* score of the central, left flanking and right flanking regions, respectively. We initially searched for footprints in the control data and for each footprint we calculated its F score_(control)_ and the F score of the equivalent region in the knockout sample, F score_(experiment)_, and the log_2_-transformed fold change of F scores (knockout/control). We also searched for new RBP interactions or sites of decreased RNase accessibility by searching for footprints in the experimental data initially, before calculating F scores and extracting the data from the equivalent region in the control sample. To estimate if the F score fold change of a footprint is significant, we built an empirical null model by shuffling the C score of both samples within the mitochondrial genome 1000 times and for each locus, calculating an F score fold change from each pair of the shuffled data sets. For the candidate footprint loci, the central footprint region was required to have a C score_(experiment)_ greater than its C score_(control)_, and the F score fold change was filtered to achieve an expected 5% false discovery rate (FDR) relative to a score obtained from random shuffling 1000 times^42^. The propensity for secondary structure was quantified according to the ratio of double-stranded RNase cleavage over single-stranded, its R score:

$$R{\rm{scor}}{{\rm{e}}_i} = {\rm{lo}}{{\rm{g}}_2}\left( {\frac{{{{\rm{A}}_{i + 1}} + 1}}{{{\rm{T}}{1_{i + 1}} + 1}}} \right)$$

Violin plots were generated with the vioplot package^43^, and the log_2_ fold change of R scores calculated as:

$${\rm{lo}}{{\rm{g}}_2}\left( {\frac{{{2^{R{\rm{scor}}{{\rm{e}}_{{\rm{KO}}}}}}}}{{{2^{R{\rm{scor}}{{\rm{e}}_{{\rm{WT}}}}}}}}} \right)$$

Control footprint analyses of the cytoplasmic 28S rRNA were carried out in an identical manner, except alignment was performed to the GENCODE vM14 reference transcript set combined with the 28S rRNA sequence downloaded from GenBank (NR_003279.1), and no significant footprints were identified (Supplementary Data 5).

### PAR-CLIP method and analyses

MEFs from *Lrpprc–FLAG* transgenic mice^20^ were maintained in DMEM medium (Thermo Fisher Scientific) supplemented with GlutaMAX-I, 10% fetal bovine serum, 1% penicillin-streptomycin and 1× MEM non-essential amino acids. HeLa Tet-On cells stably transformed with pTRE-hLRPPRC-FLAG^20^ were maintained in DMEM medium supplemented with GlutaMAX-I, 10% fetal bovine serum, 1% penicillin-streptomycin and 200 μg ml^−1^ hygromycin^44^. All cells were tested and found to be free of mycoplasma. Induction of LRPPRC-FLAG expression was achieved by addition of 2 μg ml^−1^ doxycycline to the incubation medium for 72 h. PAR-CLIP was performed according to Spitzer et al.^45^, with the exception that cells were lysed in lysis buffer (10 mM Tris-HCl (pH 7.5), 260 mM sucrose, 100 mM KCl, 20 mM MgCl_2_, 1% digitonin) in the presence of RNase inhibitor (New England Biolabs) and protease inhibitors (Roche). Lysates were clarified by centrifugation and diluted in lysis buffer without digitonin until the final digitonin concentration was 0.2%. Diluted lysates were incubated with anti-FLAG M2 magnetic beads for 2.5 h at 4 °C. Beads were washed in lysis buffer with 0.1% digitonin and then in lysis buffer without digitonin. RNA library preparation and sequencing was performed by Vertis Biotechnologie (Freising, Germany) using unique molecular identifiers to identify PCR duplicates by incorporating 10 random bases in the 5′-adapter, according to Kivioja et al.^46^.

The PAR-CLIP libraries were prepared using indexed adapter containing an 8 bp barcode followed by 8 random bases to allow multiplexing and unambiguous detection of PCR duplicates. Following de-multiplexing and de-duplication, adapter sequences were removed from PAR-CLIP reads with cutadapt^47^ v1.10, requiring a minimum length after trimming of 14 nt. As PAR-CLIP libraries detect cross-linked regions utilizing photoactivatable ribonucleosides, the reads will contain a large number of sequence mismatches relative to the reference. To prevent misalignment of mitochondrial reads to nuclear mitochondrial sequences, the mouse and human genome reference sequence (mm10 and hg38) was masked for these regions based on the mm9 and hg19 NumtS tracks from UCSC, with coordinates converted to mm10 and hg38 with UCSC liftOver^48^. Trimmed reads were aligned to the Numts-masked genome sequence with Bowtie^49^ v1.12 (–k 1 –n 2 –M 100 –chunkmbs 512 –best –strata). Cross-linked regions were identified with BMix^50^, using default parameters (minimum coverage of 5, refinement coverage of 1, a minimum posterior probability of cross-linking of 95% and strand-specific parameter calculation). MEME^51^ 4.11.2 was used to search for ungapped motifs within the binding sites identified by BMix (–rna –time 18,000 –maxsize 60,000 –mod anr –nmotifs 5 –minw 6 –maxw 50). Strand-specific coverage profiles were generated with BEDtools v2.26.0^41^. To assess the regions of the mitochondrial genome that were found to be overlapped by both PAR-CLIP and RNase footprinting, the mitochondrial genome was binned into 50 nt windows with BEDtools v2.26.0^41^. The number of PAR-CLIP sites or RNase footprints overlapping each region was calculated and the significance of the number of sites overlapped by both was tested with a hypergeometic test.

### Protein purification

Recombinant human and mouse LRPPRC and SLIRP proteins were expressed and purified as follows:^32^. Codon-optimized ORFs corresponding to the mature forms of human and mouse LRPPRC or SLIRP were expressed in Rosetta 2 cells (EMD chemicals) by induction with 0.5 mM isopropyl-1-thio-β-D-galactopyranoside (IPTG) at 30 °C for 16 h in Enpresso B media (Biosilta). After lysis, the proteins were purified over a His-Select Ni^2+^ (Sigma-Aldrich) resin and dialyzed against H-0.2 (25 mM Tris-HCl pH 7.8, 0.5 mM EDTA, 10% glycerol, 1 mM dithiothreitol, 200 mM NaCl) after the addition of TEV protease at a 1:50 protease:protein ratio. Further purification was conducted over a heparin column equilibrated in H-0.2. After washing with H-0.2, the proteins were eluted with H-0.2 buffer containing 600 mM NaCl and purified to homogeneity over a HiLoad 16/60 Superdex 200 gel filtration column (GE Healthcare) in buffer H-0.2 lacking glycerol.

### In vitro RNA-binding assays

RNA electrophoretic mobility shift assays were performed as follows:^32^. Purified proteins were incubated at room temperature for 30 min with fluorescein labeled RNA oligonucleotides (Dharmacon) in 10 mM HEPES (pH 8.0), 1 mM EDTA, 50 mM KCl, 2 mM DTT, 0.1 mg ml^−1^ fatty acid-free BSA, and 0.02% Tween-20. Reactions were analyzed by 10% PAGE in TAE and fluorescence was detected using a Typhoon FLA 9500 biomolecular imager (GE Lifesciences). RNA chaperone activity was assayed according to Rajkowitsch et al.^51^. Briefly, the following oligoribonucleotide 21R+: 5′-Cy3-AUGUGGAAAAUCUCUAGCAGU-3′ and its complement 21R−: 5′-Cy5-ACUGCUAGAGAUUUUCCACAU-3′ (Dharmacon) were hybridized in the presence or absence of 1 μM of mouse LRPPRC–SLIRP complex. Aliquots were removed at specific time points, mixed on ice with stop buffer and separated on a 20% native polyacrylamide gel at 4 °C. Fluorescence was detected with a Typhoon FLA 9500 biomolecular imager (GE Lifesciences).

### Capture of polyadenylated mRNA ends by PCR

Polyadenylation of *mt-Nd1*, *mt-Cyb* and *mt-Nd2* was examined using a modified 3′-RACE method^52^, where the Terminator nuclease treatment was omitted, since mitochondrial RNAs are not capped, and a universal or poly(A)-specific anchor primers were used in place of the “U-select primer”, since no poly(U) polymerase was present in our samples. The following gene-specific primers were used to identify the lengths of 3′-end polyadenylated products from *mt-Nd1*: 5′-GCGGGAGTACCACCATACAT-3′; *mt-Nd2*: 5′-TCCACCCTAGCTATCATAAGCA-3′; and *mt-Cyb*: 5′-GCCAACTAGCCTCCATCTCA-3′.

### RNA-Seq analyses

RNA sequencing was performed on total RNA from four control and four *Lrpprc* knockout mouse hearts on an Illumina HiSeq platform, according to the Illumina Tru-Seq protocol. We used random hexamer primers for cDNA library generation and carried out cytoplasmic rRNA depletion using the Ribo-Zero rRNA removal kit. Sequenced reads were aligned against the mouse transcriptome with RSEM v1.2.31 (–bowtie2 –strandedness reverse –paired-end) using the GENCODE vM13 transcript set combined with custom mitochondrial rRNA and mRNA sequences with an additional 50 adenine residues added to the 3′-ends to allow mapping of polyA tails. Gene-level counts were imported and analyzed for differential expression with tximport v1.4.0^53^ and DESeq2 v1.16.1. Coverage profiles for each mitochondrial transcript were generated from full fragment BED files with BEDtools v2.26.0^41^.

### Data availability

All the data needed to evaluate the conclusions in the paper are present in the paper and/or the Supplementary Information. All the sequencing data generated in this study are available at the NCBI Gene Expression Omnibus (GEO, accession number GSE100733). The additional data related to this paper may be requested from the authors.

## Electronic supplementary material

Supplementary Information

Description of Additional Supplementary Files

Supplementary Data 1

Supplementary Data 2

Supplementary Data 3

Supplementary Data 4

Supplementary Data 5
