# Supplementary material for: LRPPRC-mediated folding of the mitochondrial transcriptome
Source: Nat Commun. 2017 Nov 16;8:1532. doi: 10.1038/s41467-017-01221-z (PMC5691074; doi:10.1038/s41467-017-01221-z)
Supplement: Supplementary file 1 — Supplementary Information [file 41467_2017_1221_MOESM1_ESM.pdf]

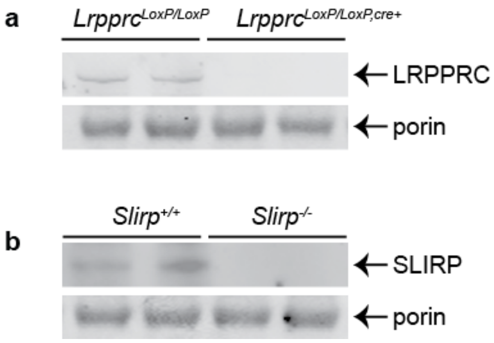

Supplementary Figure 1 | Immunoblots showing that LRPPRC is lost in heart mitochondria from *Lrpprc* conditional heart and skeletal-knockout, *Lrpprc*$^{loxP/loxP,cre+}$, mice compared to control *Lrpprc*$^{loxP/loxP}$ mice (**a**) and SLIRP is lost in heart mitochondria from *Slirp* full body knockout mice, *Slirp*$^{-/-}$, compared to control, *Slirp*$^{+/+}$, mice (**b**). Porin was used as a loading control for each immunoblot.

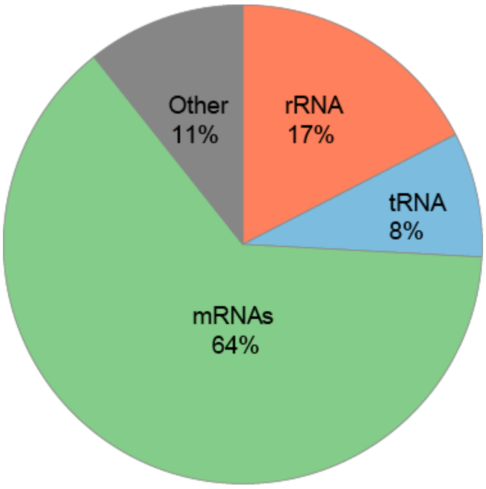

Supplementary Figure 2 | Location of LRPPRC footprints in different classes of mt-RNAs.

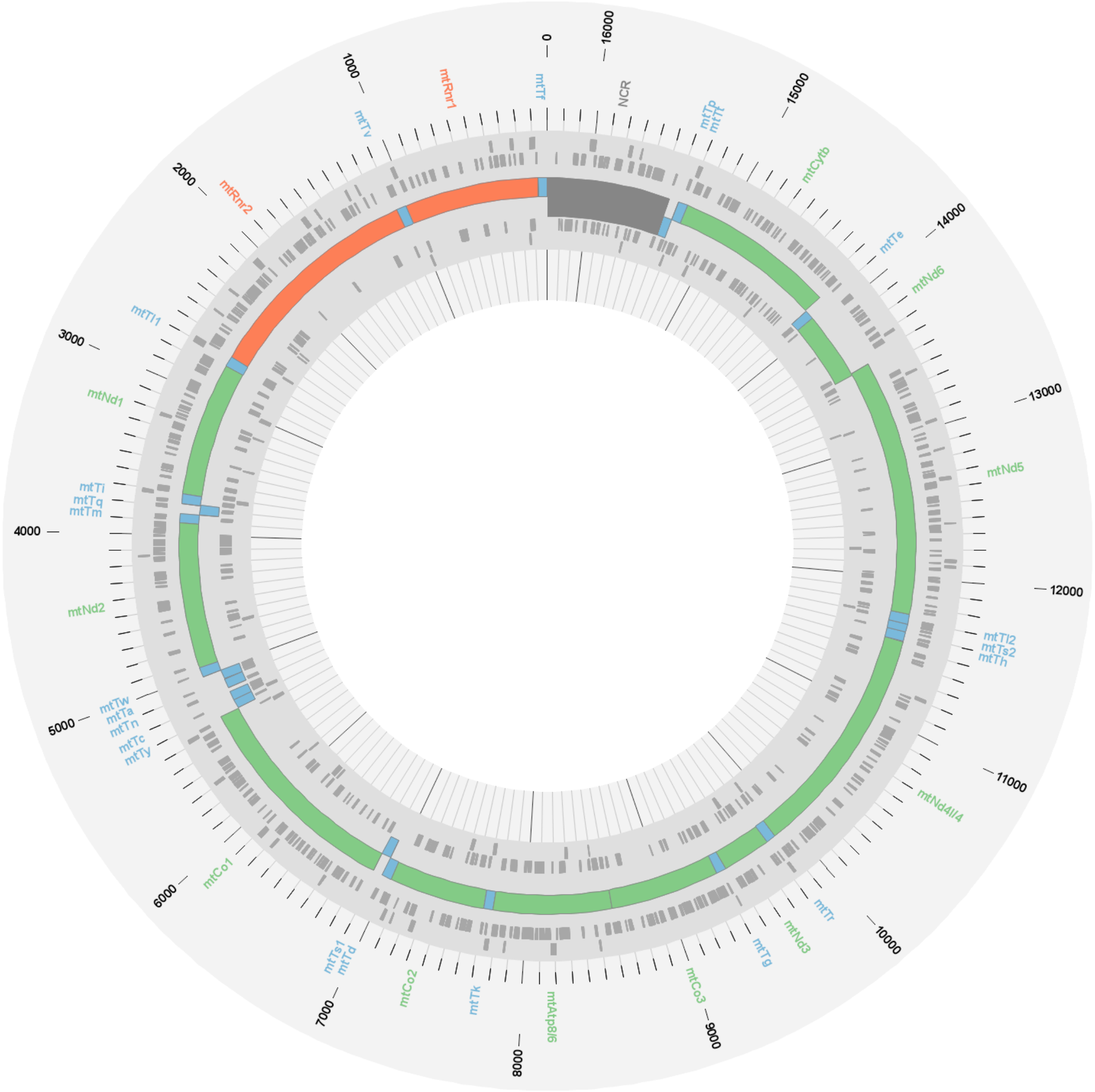

Supplementary Figure 3 | SLIRP does not bind mitochondrial RNAs *in vivo*.
Circular representation of the mitochondrial genome (centre track) displaying the putative footprint sites (grey bars) that showed differences in RNase protection between control and *Slirp* knockout mice, but these changes were not significant at a false discovery rate of ≤ 0.05.

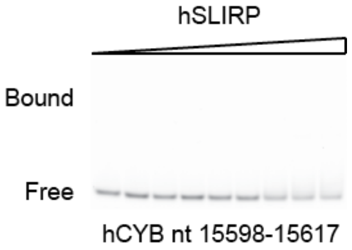

Supplementary Figure 4 | Human SLIRP does not bind RNA *in vitro*. RNA EMSA of human SLIRP with hCYB nt 15598-15617 target RNA.

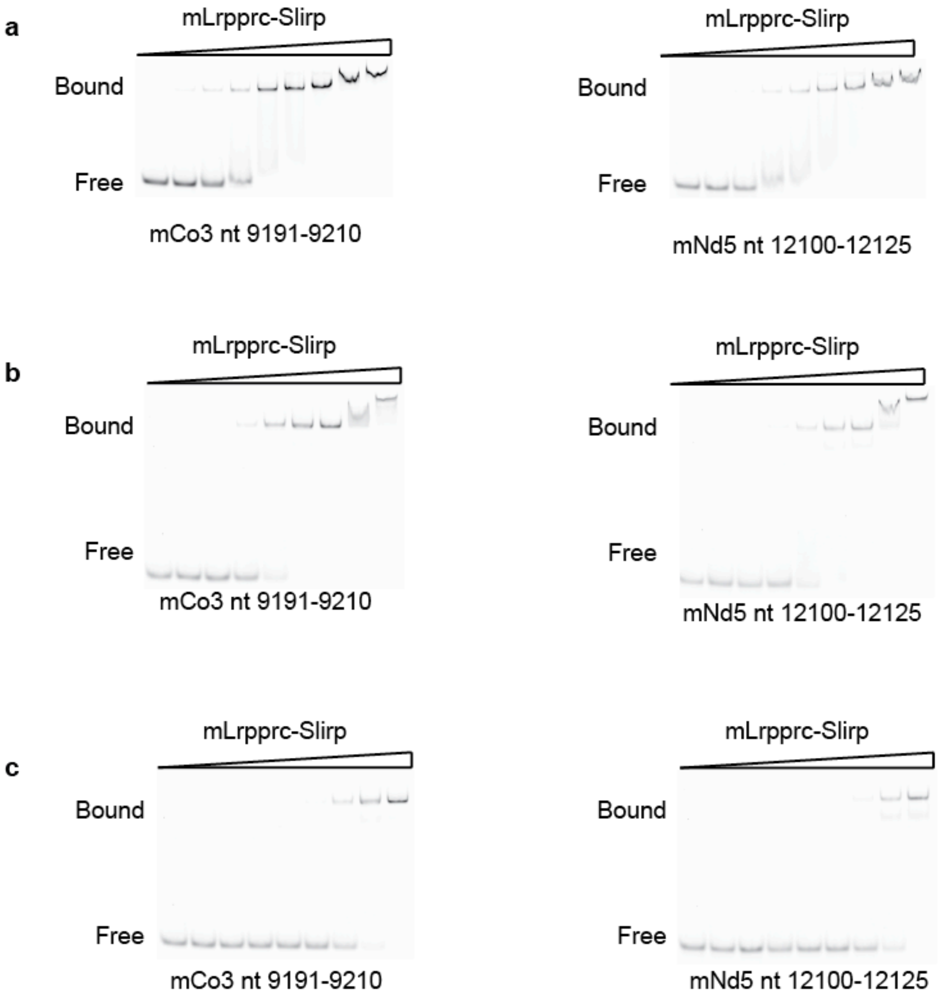

Supplementary Figure 5 | LRPPRC-SLIRP competitor assay.
RNA EMSA of the mouse LRPPRC-SLIRP complex with mCo3 nt 9191-9210 target RNA (left panel) and mNd5 nt 12100-12125 non-target RNA (right panel). The RNA EMSAs were performed in the absence (**a**) or presence of 10x (**b**) and 100x (**c**) molar excess of unlabeled mNd5 12840-12865 non-target mRNA.

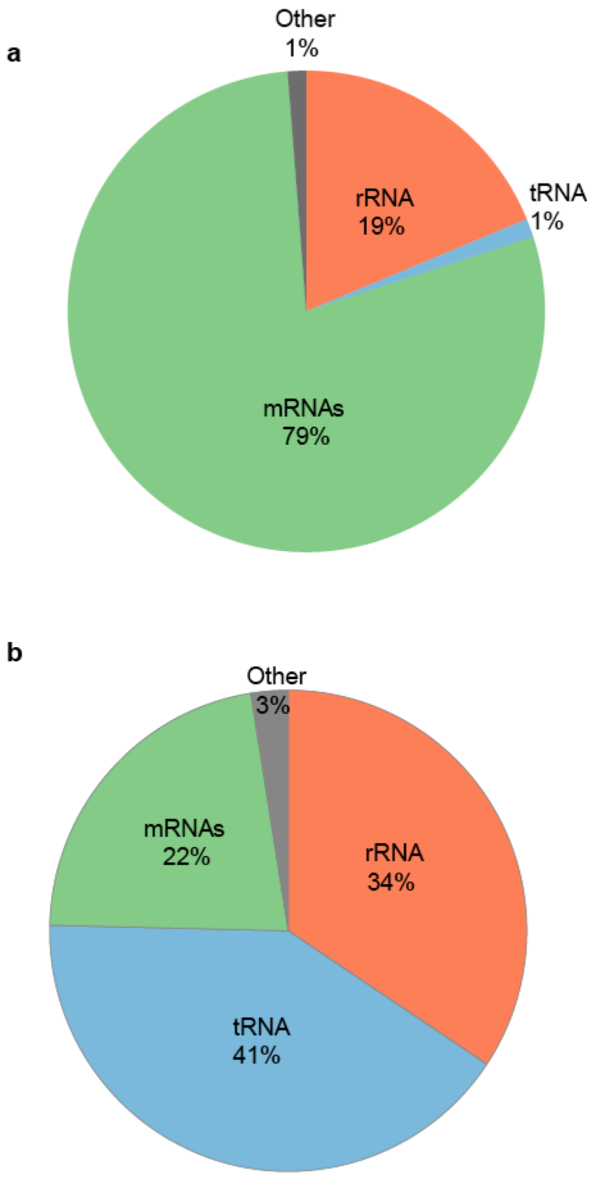

Supplementary Figure 6 | (**a**) Location of high confidence LRPPRC binding sites in different classes of mt-RNAs, determined by overlapping identified footprints and PAR-CLIP binding sites; and locations footprints that do not overlap with PAR-CLIP binding, normalized to transcript length (**b**).

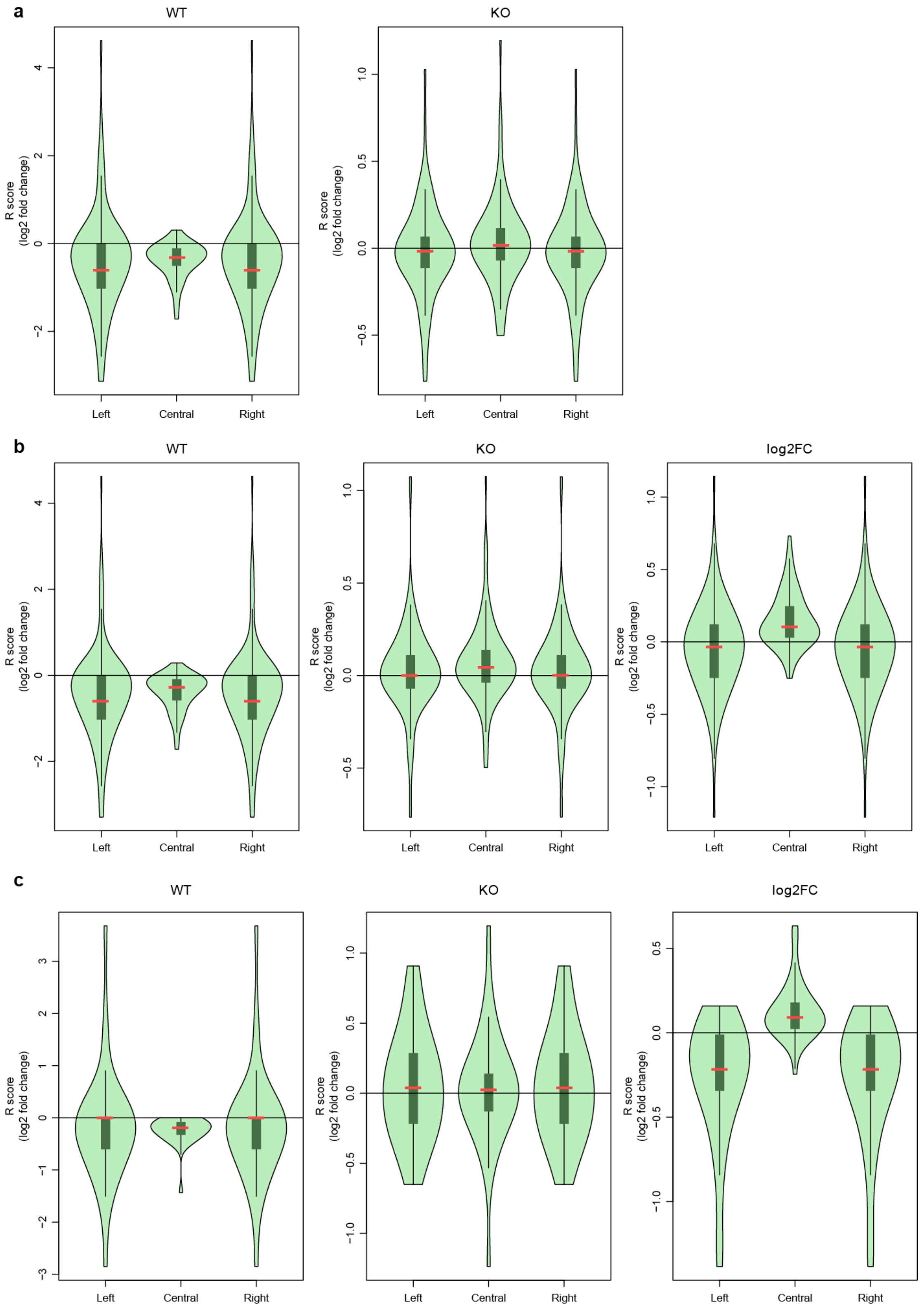

Supplementary Figure 7 | LRPPRC is necessary to relax secondary structures of mitochondrial RNAs to enable their maturation and polyadenylation.

(**a**) The distribution of average R scores across the left flanking, central footprint and right flanking regions (10 nt) of all footprints that overlap PAR-CLIP binding sites identified by BMix, in wild-type and knockout mice.

(**b**) The distribution of average R scores and changes in R score in mRNA-overlapping footprints. The average R score across the left flanking, central footprint and right flanking regions of all footprints that overlap mitochondrial mRNA coding sequences, in wild-type and knockout mice. The average $\log_2$ fold change of the R score across the left flanking, central footprint and right flanking regions (10 nt) of all footprints that overlap mitochondrial mRNA-encoding regions.
(**c**) The distribution of average R scores and changes in R score in rRNA-overlapping footprints. The average R score across the left flanking, central footprint and right flanking regions (10 nt) of all footprints that overlap mitochondrial rRNA coding sequences, in wild-type and knockout mice. The average $\log_2$ fold change of the R score across the left flanking, central footprint and right flanking regions of all footprints that overlap mitochondrial rRNA-encoding regions.

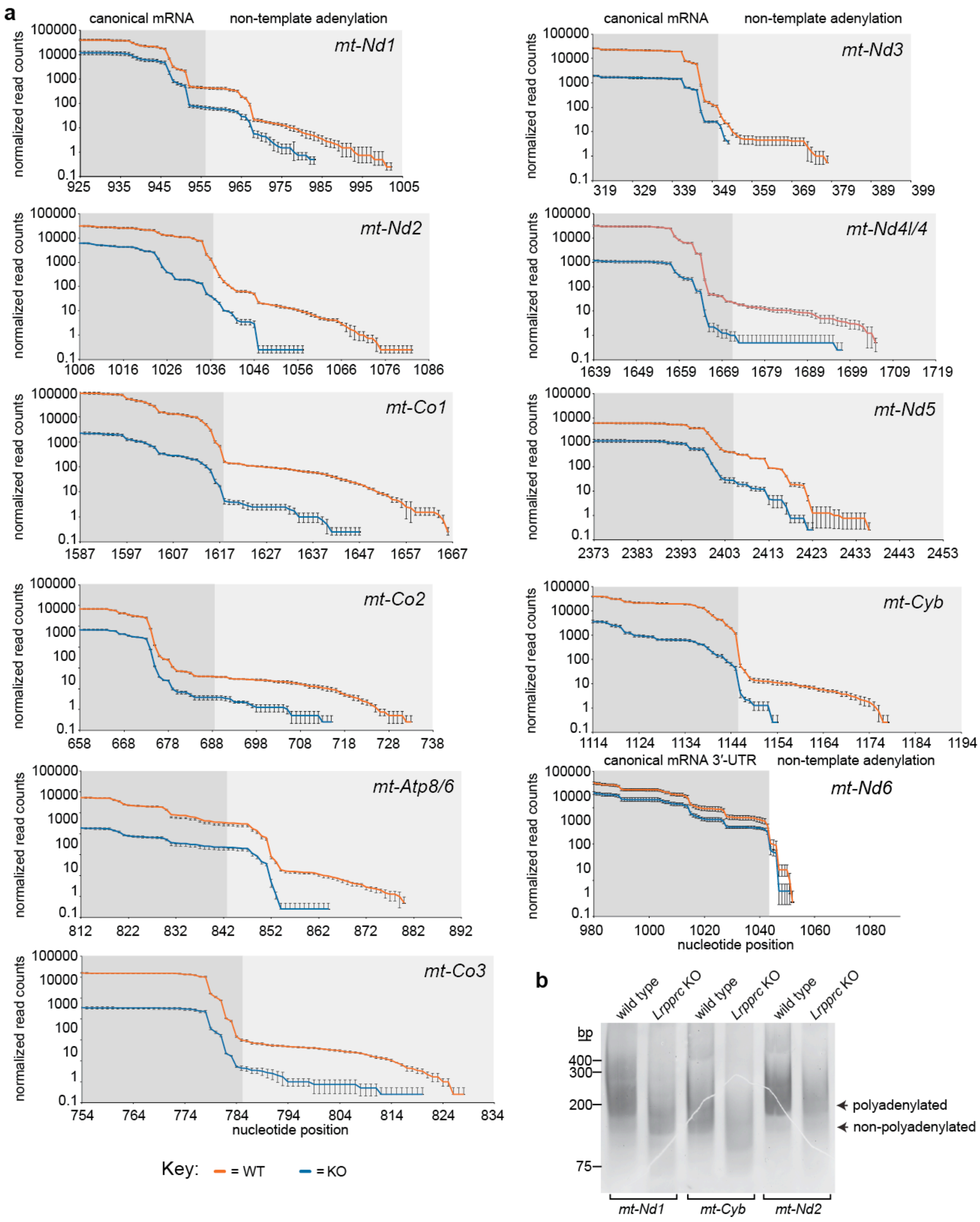

Supplementary Figure 8 | LRPPRC is required for polyadenylation of mitochondrial mRNAs.

(**a**) Mapping of RNA-Seq reads to polyadenylated reference sequences reveals a reduction in polyadenylation in *Lrpprc* knockout hearts (blue line), compared to wild-type hearts (orange line). The canonical 3′ region of each mRNA is shown in dark grey and the section of polyadenosine is shown in light grey.

(**b**) A modified 3′-RACE method confirms the reduced length of the 3′ poly(A) tails of *mt-Nd1*, *mt-Nd2* and *mt-Cyb* mRNAs.

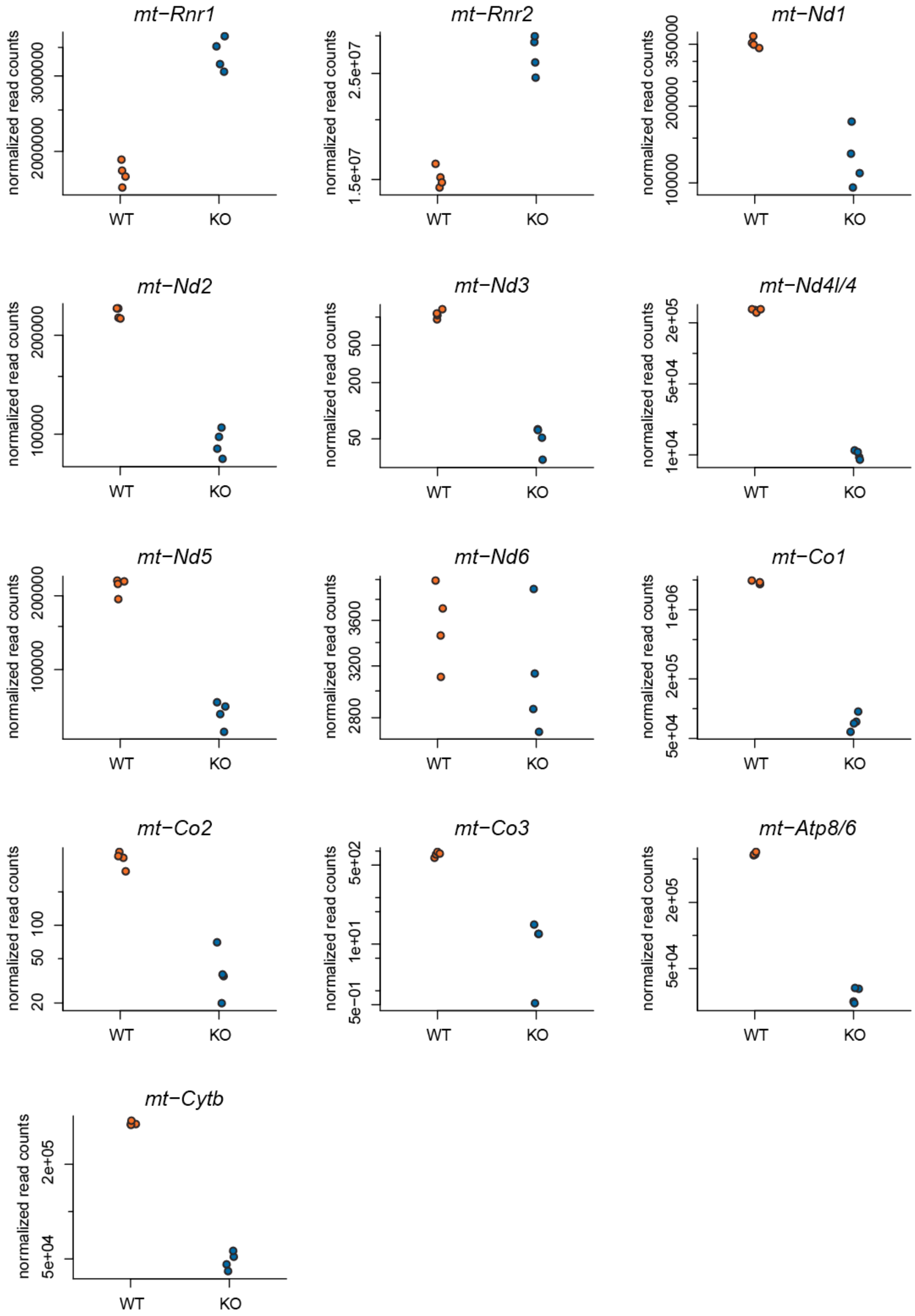

Supplementary Figure 9 | Loss of LRPPRC chaperone activity leads to significantly reduced stability of mitochondrial mRNAs, identified by DE-Seq analyses of RNA-Seq datasets from four control and four *Lrpprc* knockout mice.

Supplementary Table 1 | PAR-CLIP library statistics for LRPPRC and control samples.

	LRPPRC (HeLa cells)	Control (HeLa cells)	LRPPRC (MEFs)	Control (MEFs)
**Total Reads**	25,911,145	2,213,917	22,189,108	2,655,345
**Mapped Reads**	13,749,342	40,557	8,596,138	58,745
**Unmapped Reads**	12,161,803	2,173,360	13,592,970	2,596,600
**Alignment Rate**	53.06%	1.83%	38.74%	2.21%

Supplementary Table 2 | High confidence binding sites of LRPPRC.

Chrom	Start	End	Name	FDR	Strand
chrM	906	943	footprints_37_0.129019_1.056814	0.0255	+
chrM	1146	1157	footprints_11_-0.009823_1.141524	0.0433	+
chrM	1177	1202	footprints_25_0.047596_0.726789	0.0313	+
chrM	1204	1214	footprints_10_0.015022_0.593249	0.0059	+
chrM	1216	1240	footprints_24_0.093546_0.654385	0.0097	+
chrM	1290	1306	footprints_16_0.046507_0.788845	0.0379	+
chrM	1308	1316	footprints_8_0.107292_0.868081	0.0081	+
chrM	1351	1360	footprints_9_0.153585_1.115471	0.0344	+
chrM	1362	1389	footprints_27_0.047183_0.693594	0.0314	+
chrM	1527	1536	footprints_9_-0.092514_0.646160	0.0028	+
chrM	1598	1613	footprints_15_-0.056706_0.580301	0.0242	+
chrM	1786	1798	footprints_12_0.000000_0.633178	0.0227	-
chrM	2209	2219	footprints_10_-0.037586_0.621145	0.0359	+
chrM	2355	2363	footprints_8_-0.123076_1.243978	0.0311	+
chrM	2457	2485	footprints_28_0.042262_0.679093	0.0074	+
chrM	2489	2513	footprints_24_-0.272451_0.412981	0.003	+
chrM	2808	2826	footprints_18_-0.020005_1.098520	0.0316	+
chrM	2910	2919	footprints_9_0.175478_1.283608	0.0356	+
chrM	3130	3148	footprints_18_-0.184633_0.898067	0.043	+
chrM	3358	3370	footprints_12_0.073606_0.852337	0.0287	+
chrM	3381	3390	footprints_9_-0.459572_0.689690	0.0335	+
chrM	3465	3473	footprints_8_0.031996_0.690022	0.0208	+
chrM	3987	4004	footprints_17_-0.336617_0.864999	0.0409	+
chrM	4057	4067	footprints_10_-0.058888_0.893090	0.0096	+
chrM	4593	4615	footprints_22_0.022590_0.970611	0.0285	+
chrM	4682	4700	footprints_18_0.042832_0.755120	0.0312	+
chrM	5400	5410	footprints_10_-0.199968_0.979841	0.0267	+
chrM	5543	5566	footprints_23_-0.159854_0.760140	0.0477	+
chrM	5623	5634	footprints_11_0.064993_0.797478	0.0142	+
chrM	5646	5666	footprints_20_0.037364_0.476687	0.0067	+
chrM	5716	5724	footprints_8_-0.255098_0.659260	0.0117	+

chrM	5735	5747	footprints_12_-0.123641_0.872596	0.0221	+
chrM	6602	6625	footprints_23_0.002798_0.858258	0.0358	+
chrM	6648	6656	footprints_8_-0.020099_0.787553	0.0188	+
chrM	6674	6683	footprints_9_-0.343342_0.769899	0.0173	+
chrM	6734	6742	footprints_8_-0.534212_0.541813	0.002	+
chrM	6790	6823	footprints_33_0.009759_0.740846	0.0147	+
chrM	7181	7199	footprints_18_0.099354_0.642739	0.0261	+
chrM	7201	7213	footprints_12_0.177927_0.733665	0.0224	+
chrM	7323	7335	footprints_12_-0.234772_0.858858	0.0369	+
chrM	7381	7391	footprints_10_-0.090922_1.044730	0.0229	+
chrM	7422	7431	footprints_9_-0.758493_0.389374	0.003	+
chrM	7495	7528	footprints_33_-0.040067_0.690882	0.0269	+
chrM	7817	7833	footprints_16_0.011332_0.984716	0.0368	+
chrM	7858	7895	footprints_37_0.029717_0.825845	0.0363	+
chrM	7896	7913	footprints_17_-0.099399_1.036558	0.0315	+
chrM	7964	7977	footprints_13_-0.294637_0.513339	0.038	+
chrM	8113	8122	footprints_9_-0.242048_0.645574	0.0057	+
chrM	8236	8276	footprints_40_-0.114556_0.733008	0.0312	+
chrM	8296	8308	footprints_12_-0.006734_0.848416	0.043	+
chrM	8384	8397	footprints_13_-0.525200_0.518012	0.003	+
chrM	8438	8446	footprints_8_-0.349717_0.590279	0.0265	+
chrM	8632	8642	footprints_10_-0.075410_0.979148	0.0408	+
chrM	8763	8783	footprints_20_-0.167548_0.598488	0.0059	+
chrM	8784	8792	footprints_8_-0.153242_1.257652	0.0356	+
chrM	8934	8942	footprints_8_-0.514955_0.686197	0.0309	+
chrM	9012	9032	footprints_20_-0.091936_0.851356	0.0404	+
chrM	9191	9210	footprints_19_-0.536715_0.473365	0.0097	+
chrM	9246	9255	footprints_9_-0.246620_0.904155	0.0407	+
chrM	9297	9308	footprints_11_-0.263529_1.072086	0.0471	+
chrM	9505	9515	footprints_10_-0.041695_0.736412	0.0074	+
chrM	10217	10232	footprints_15_0.001171_0.939822	0.0233	+
chrM	10348	10365	footprints_17_0.052462_0.952521	0.0403	+
chrM	10578	10588	footprints_10_-0.321613_0.806516	0.0314	+
chrM	10662	10691	footprints_29_-	0.0465	+

			0.005570_0.949365		
chrM	11147	11174	footprints_27_-0.074368_0.554777	0.0083	+
chrM	11178	11218	footprints_40_-0.096406_0.584476	0.0111	+
chrM	11406	11414	footprints_8_-0.142880_0.988735	0.034	+
chrM	11699	11720	footprints_21_-0.145324_1.199560	0.0402	+
chrM	11988	11996	footprints_8_0.153161_1.246775	0.0402	+
chrM	12567	12582	footprints_15_-0.287605_0.852714	0.0368	+
chrM	12597	12608	footprints_11_0.049257_1.332191	0.0376	+
chrM	13385	13419	footprints_34_-0.039015_0.819830	0.0262	+
chrM	13770	13799	footprints_29_0.074552_0.825320	0.0192	+
chrM	13824	13846	footprints_22_-0.032980_0.754464	0.0142	+
chrM	14233	14242	footprints_9_-0.442847_0.815809	0.0254	+
chrM	14337	14368	footprints_31_-0.111271_0.826867	0.0229	+
chrM	14638	14672	footprints_34_0.014980_0.680558	0.0076	+
chrM	14995	15015	footprints_20_-0.227673_0.634789	0.0195	+
chrM	15051	15065	footprints_14_0.050168_1.013145	0.0327	+
