# Supplementary material for: LRPPRC-mediated folding of the mitochondrial transcriptome
Source: Nat Commun. 2017 Nov 16;8:1532. doi: 10.1038/s41467-017-01221-z (PMC5691074; doi:10.1038/s41467-017-01221-z)
Supplement: Supplementary file 2 — Description of Additional Supplementary Files [file 41467_2017_1221_MOESM2_ESM.pdf]

Description of Additional Supplementary Files

File Name: Supplementary Data 1
Description: WT called LRPPRC footprints with statistically significant increases in F score (indicating a reduction in footprint depth in the KO).

File Name: Supplementary Data 2
Description: WT called SLIRP footprints with no statistically significant increases in F score.

File Name: Supplementary Data 3
Description: KO called LRPPRC footprints with statistically significant increase in F score (indicating a increase in footprint depth in the KO).

File Name: Supplementary Data 4
Description: Average R scores across rRNA- and mRNA-encoding regions in LRPPRC and SLIRP WT and KO samples.

File Name: Supplementary Data 5
Description: Control footprint analyses of the cytoplasmic 28S rRNA in LRPPRC WT and KO samples.
